# Understanding the rationale for metronidazole use in dogs and cats

**DOI:** 10.1111/jsap.13910

**Published:** 2025-06-30

**Authors:** J. Ng, N. Steffensen, I. Battersby, J. S. Weese, D. Timofte, P. L. Toutain, J. L. Granick, J. Elliott, S. Choi, T. Sparks, S. Tavener, F. Allerton

**Affiliations:** ^1^ Department of Internal Medicine Davies Veterinary Specialists, part of Linnaeus Veterinary Limited Higham Gobion UK; ^2^ Department of Internal Medicine Willows Veterinary Centre & Referral Service Solihull UK; ^3^ Department of Small Animal Medicine and Surgery University of Veterinary Medicine, Hannover, Foundation Hannover Germany; ^4^ Mars Veterinary Health Vancouver Washington USA; ^5^ Centre for Public Health and Zoonoses Ontario Veterinary College – University of Guelph Guelph Ontario Canada; ^6^ Institute of Infection, Veterinary and Ecological Sciences University of Liverpool Neston UK; ^7^ Department of Comparative Biomedical Sciences The Royal Veterinary College London UK; ^8^ Department of Veterinary Clinical Sciences University of Minnesota College of Veterinary Medicine Saint Paul Minnesota USA; ^9^ Waltham Petcare Science Institute Melton Mowbray UK; ^10^ Highcliff Veterinary Practice Limited Ipswich UK

## Abstract

**Objective:**

It is currently unknown how often antibiotics (including metronidazole) are used for non‐antibacterial purposes in dogs and cats. This study looked to characterise the rationale for metronidazole prescription in these species.

**Methods:**

Retrospective cohort study. Veterinarians reported clinical information for dogs and cats treated with metronidazole in the previous year, including the rationale for metronidazole selection.

**Results:**

Three hundred and thirty‐two cases were reported by 138 veterinarians describing metronidazole use in 47 cats and 285 dogs. Metronidazole was most commonly prescribed to treat acute diarrhoea (*n* = 156, 47%), chronic diarrhoea (*n* = 79, 24%) or giardiasis (*n* = 36, 11%). Veterinarians selected metronidazole exclusively for non‐antimicrobial targeted therapy in 42% of cases (125/300). Putative anti‐inflammatory/immunomodulatory properties were cited in 64% of cases (213/332). Educational resources (41/92, 45%), team‐based collaboration (29/92, 32%) and specialist consultation (10/92, 11%) were cited as the supportive basis for these prescription choices.

**Clinical significance:**

Veterinarians are using metronidazole frequently for non‐antimicrobial properties in contradiction to antimicrobial use guidelines. Future stewardship programs should adapt guidance specifically to counter this prescribing behaviour.

## INTRODUCTION

Almost 5 million human deaths were associated with bacterial antimicrobial resistance (AMR) in 2019 (Murray et al., 2022) and the situation is predicted to get worse (O’Neill, 2016). The health and welfare of cats and dogs may also be negatively impacted by AMR (Santana et al., 2023; Weese, 2008). Furthermore, since pets can act as reservoirs for zoonotic transfer of resistant bacteria or mechanisms of resistance (Belas et al., 2020; Drougka et al., 2016; Kaspar et al., 2018; Pomba et al., 2017), veterinarians have an important role to play in a One Health approach to tackling AMR. To this end, various antimicrobial stewardship resources, including recommendations for rational antimicrobial use (AMU) have been developed (Allerton et al., 2021). A consistent theme across the different AMU guidelines is the recommendations not to use antimicrobials for the management of acute and chronic diarrhoea unless the animal is showing signs of sepsis or following appropriate dietary trials, the patient exhibits signs of true primary infection (such as systemic inflammatory response syndrome or evidence of adherent‐invasive bacteria) (Allerton et al., 2021; Cerquetella et al., 2020; Jessen et al., 2024).

However, despite this guidance, AMU was reported in 28 to 70% of canine diarrhoea cases in the United Kingdom (Anholt et al., 2014; Fins et al., 2023; Pegram et al., 2023; Singleton et al., 2017). Of these, metronidazole is the most commonly prescribed antimicrobial for gastrointestinal disease, selected in 47 to 65% of canine diarrhoea cases (Pegram et al., 2023; Singleton et al., 2019). Metronidazole, a nitroimidazole antibacterial and antiprotozoal agent, is included in the World Small Animal Veterinary Association’s list of essential medications (Steagall et al., 2023). It is indicated to treat microaerophilic and anaerobic microorganisms only, since its activity is dependent on prior reduction to short‐lived toxic intermediates in a low oxygen environment (Leitsch, 2019). Putative immunomodulatory or anti‐inflammatory activity may also drive the selection of metronidazole for the treatment of chronic enteropathy (Ellis et al., 2023; Makielski et al., 2019) although the evidence base for this mechanism of action is, at best, equivocal (Ellis et al., 2023). Moreover, metronidazole use in dogs can negatively impact the gut microbiome and metabolome (Igarashi et al., 2014; Pilla & Suchodolski, 2020), can cause serious neurotoxicity, even at standard doses (Tauro et al., 2018) and in humans, its use may increase the likelihood of developing chronic enteropathy at a later date (Faye et al., 2023).

The reasons underlying this discordance between AMU recommendations and prescribing habits in practice have been explored from a behavioural science standpoint (Tompson et al., 2021) but remain largely unknown. The primary objective of this study was to explore whether there are reasons or beliefs regarding the clinical indications for metronidazole use in dogs and cats that have not been previously investigated. The study aimed to determine the proportion of metronidazole use that was for non‐antimicrobial purposes and to compare outcome measures based on the stated rationale for use. Improved understanding of veterinary prescribing behaviour could help guideline makers improve stewardship interventions. In the future, educational resources targeted to specific prescriber rationales for use of antimicrobials could help minimise their unnecessary use.

## METHODS

This study was a retrospective case series including a survey component to determine the clinician’s rationale for metronidazole use. Ethical approval was obtained from the Royal College of Veterinary Surgeons (023‐098‐Ng). The raw data were stored as anonymised data. No personal data that could allow for the identification of any of the animals or participant were collected.

### Inclusion criteria

Over 10,000 veterinarians were invited to participate in a study regarding metronidazole use in dogs and cats. The study was disseminated to veterinarians working in primary care, referral, university, shelter or charity practices across Canada, France, Germany, Singapore, the United Kingdom and the United States. The invitation was distributed via a survey link shared through various channels including the Deutsche Veterinärmedizinische Gesellschaft, European Network for Optimization of Veterinary Antimicrobial Therapy, L’association Française des Vétérinaires pour Animaux de Compagnie, Royal College of Veterinary Surgeons practice data, Singapore Veterinary Association and the Small Animal Medicine Society. Consent from the participant was required prior to data entry on an electronic data capture platform (CastorEDC®).

### Study design

The study was performed in two phases. In both phases, participants were asked questions about the five most recent cases they had treated with metronidazole (all must have been seen within the previous calendar year to ensure accurate recall). In phase I, participants were asked to provide a rationale for metronidazole use for each case, via an open question style. The answers of phase I were collated and thematically grouped by the primary investigators. This was performed on the first 50 cases in the order generated by CastorEDC® (grouped by veterinarian). The goal of the phase I survey was to recruit sufficient participants (and cases) to reach theoretical saturation regarding the rationale for metronidazole use. Theoretical saturation was achieved when no new selection factors emerged from 10 consecutive cases. In phase II the metronidazole rationale question was posed in a closed multiple choice format (with multi‐select options) to a different set of participants.

### Outcomes

In both phases, information was collected regarding animal signalment (species, breed, age and bodyweight); condition treated (clinician diagnosis); investigations performed to support the diagnosis; metronidazole details (dose and frequency); rationale for metronidazole selection and treatment outcome. Some veterinarian characteristics (type of practice, country of practice/education and career start date) were also collected. In phase II, participants that cited anti‐inflammatory/immunomodulatory as the rationale for metronidazole use were asked additional questions relating to the source of this proposed mechanism and whether they use other antibiotics in this manner.

As participants could cite multiple rationales for their selection of metronidazole, all instances where the rationale was either “anti‐bacterial” or “anti‐protozoal” were categorised as antimicrobial. Exclusions from further analysis included those that solely cited anti‐diarrhoeal action (since this includes both antimicrobial and non‐antimicrobial targeted therapy rationales) and those that did not provide a rationale for their selection of metronidazole.

### Statistical analysis

Statistical analysis was primarily descriptive. The primary outcome of interest related to the rationale cited for metronidazole selection and the source of evidence of anti‐inflammatory/immunomodulatory rationales. Binary and categorical data were summarised by frequency and percentages and compared using chi‐square tests of association or Fisher exact tests if counts were low. In the case of missing responses, percentages are calculated over available data. Continuous data were summarised by median and interquartile range (IQR) and compared using Mann–Whitney tests adjusted for ties. All data were analysed using commercially available statistical software (SPSSxx and Minitab21).

## RESULTS

### Respondent characteristics/demographics

One hundred and thirty‐eight veterinarians participated in the study with a median of 1 case (IQR 1 to 5) reported per veterinarian and a total of 332 cases. These included 285 dogs and 47 cats treated with metronidazole. Most veterinarians worked (110/138, 80%) in the United Kingdom. Non‐UK participants were geographically diverse, including those working in Finland (*n* = 3, 2%), Germany (*n* = 6, 4%), North Macedonia (*n* = 2, 1%), Singapore (*n* = 4, 3%) or the United States (*n* = 5, 4%). Other countries veterinarians worked in (each *n* = 1, 1%) included Austria, Belgium, Canada, Indonesia, Portugal, Spain and Sweden. Similarly, most veterinarians graduated from institutions in the United Kingdom (72/115, 63%). Others graduated from institutions in Australia (*n* = 2, 2%), the Czech Republic (*n* = 2, 2%), Finland (*n* = 2, 2%), Germany (*n* = 3, 3%), Italy (*n* = 4, 3%), North Macedonia (*n* = 2, 2%), Poland (*n* = 3, 3%), Portugal (*n* = 2, 2%), South Africa (*n* = 4, 3%), Spain (*n* = 5, 4%) and the United States (*n* = 3, 3%). Other countries with graduates (each *n* = 1, 1%) included Belgium, Bulgaria, Greece, Grenada, Ireland, Japan, Romania, Slovakia, Slovenia, St. Kitts and Nevis and Zimbabwe. The majority (87/118, 74%) of veterinarians worked in primary care small animal‐only practice. Other types of practice included referral practice/university (19/118, 16%), charity (6/118, 5%) and primary care mixed practice (6/118, 5%).

Sixty‐two different dog breeds were represented, including 46 (16%) crossbreeds, 32 (12%) Labrador Retrievers, 15 (6%) English Cocker Spaniels, 14 (5%) Golden Retrievers, 12 (4%) Jack Russell Terriers, 10 (4%) Border Collies, 9 (3%) Yorkshire Terriers, 8 (3%) English Springer Spaniels and 7 (2%) each of Border Terriers, German Shepherd Dogs, Miniature Schnauzers, Shih Tzu and West Highland White Terriers. Nine different cat breeds were represented, including 25 (54%) Domestic Shorthairs, 5 (11%) Maine Coons and 5 (11%) Ragdolls. Other breeds are listed in the supplementary materials (Tables S1 and S2).

### Conditions treated and diagnostic testing

Metronidazole was prescribed most frequently for the treatment of acute diarrhoea (*n* = 156, 47%), chronic diarrhoea (*n* = 79, 24%) or giardiasis (*n* = 36, 11%). Among animals receiving metronidazole, infectious disease testing was pursued in 128/332 (39%) cases. This included faecal bacteriology/parasitology (*n* = 82, 25%), culture of non‐faecal samples (*n* = 34, 10%), cytologic evaluation (*n* = 21, 6%), histopathologic evaluation (*n* = 19, 6%), polymerase chain reaction (*n* = 6, 2%) and faecal enterotoxin assays (*n* = 3, 1%). Table 1 summarises the conditions reported and the proportion of those cases in which no infectious disease tests were performed. Table 2 summarises the cases in which faecal bacteriology/parasitology and cytologic evaluation were performed in acute or chronic diarrhoea cases.

**Table 1:** Conditions treated with metronidazole and the number of cases without infectious disease testing

Condition	Number of cases	Infectious disease tests not performed
Acute diarrhoea	156	116 (75%)
Chronic diarrhoea*	79	26 (33%)
Giardiasis	36	4 (11%)
Pancreatitis	16	13 (81%)
Gastrointestinal surgery	15	12 (80%)
Bite or traumatic wounds	14	6 (43%)
Bacteraemia/sepsis	10	8 (80%)
Septic peritonitis	9	3 (33%)
Skin infection	8	3 (38%)
Anal gland abscessation	7	4 (57%)
Cholangitis/cholangiohepatitis	5	1 (20%)
Hepatic encephalopathy	5	4 (80%)
Internal abscessation	5	1 (20%)
Pyothorax	4	0 (0%)
Tetanus	4	2 (50%)
Dental disease	3	2 (67%)
Otitis	2	0 (0%)
Oral infection	2	1 (50%)
Orbital abscessation	2	2 (100%)

* Three cases reported with both acute and chronic diarrhoea were classified under chronic diarrhoea

**Table 2:** Faecal bacteriology/parasitology and cytologic evaluation performed in acute or chronic diarrhea

Condition	Faecal bacteriology/parasitology	Cytologic evaluation
Acute diarrhoea (156 cases)	38 (24%)	4 (3%)*
Chronic diarrhoea (79 cases)	42 (53%)	6 (8%)^†^

* Three cases did both cytologic evaluation and faecal bacteriology/parasitology

^†^ One case did both cytologic evaluation and faecal bacteriology/parasitology

### Rationales for metronidazole use

The answers provided in phase I for the rationale behind metronidazole use were collated and grouped into the following categories: anti‐inflammatory/immunomodulatory properties, anti‐diarrheal action, owner or clinic/practice expectations, suspected/confirmed anaerobic or susceptible bacterial infection, treatment for protozoal infections, patient systemically unwell/severe disease and previous positive outcome in a similar case or the same patient.

In phase II, cases in which the rationale for metronidazole use was cited as anti‐diarrhoeal (*n* = 92, 38%) were reclassified into specific anti‐diarrhoeal effects as follows: anti‐inflammatory/immunomodulatory properties (*n* = 65, 71%), modulation of the bacterial microbiota (*n* = 47, 51%), anti‐protozoal activity (*n* = 34, 37%) and effects on pathogenic bacteria (*n* = 23, 25%). Table 3 lists the frequency of rationales cited for metronidazole use.

**Table 3:** Summary of rationales for metronidazole use

Rationale	Phase I (91)	Phase II (241)	Total (332)
Anti‐inflammatory/immunomodulatory properties (including effects on the bacterial microbiota)	10 (11%)	203 (84%)	213 (64%)
Suspected/confirmed anaerobic/susceptible bacterial infection	32 (35%)	93 (39%)	125 (38%)
Patient systemically unwell/severe disease	26 (29%)	60 (25%)	86 (26%)
Treatment for protozoal infections	7 (8%)	78 (32%)	85 (26%)
Previous positive outcome in similar case/same patient	12 (13%)	52 (22%)	64 (19%)
Anti‐diarrhoeal action	38 (42%)	Recategorized	38 (NA)
Owner or clinic/practice expectations	11 (12%)	19 (8%)	30 (9%)

Veterinarians selected metronidazole for exclusively non‐antimicrobial targeted therapy in 42% of cases (125/300). Additionally, 74% (221/300) included at least one non‐antimicrobial targeted therapy rationale as justification for metronidazole selection. Data from 32 (10%) animals were excluded from further analysis: 8 from phase I that solely cited anti‐diarrhoeal action and 24 (7%) that did not provide a rationale for their selection of metronidazole.

### Analysis

There were both age and species‐related risk factors for metronidazole use; there was a significantly higher percentage of dogs (114/258, 44%) than cats (11/42, 26%) in which metronidazole was used for non‐antimicrobial targeted therapy (P = 0.028). Animals were significantly older (P < 0.001) when metronidazole was used for non‐antimicrobial targeted therapy (median 7 years, IQR 2 to 11) compared to use for antimicrobial reasons (median 4 years, IQR 1 to 8). Metronidazole use was not significantly influenced by breed.

The largest proportion (46/116, 40%) of study participants had graduated between 2010 and 2019 (Table 4). The proportions of veterinarians selecting metronidazole for non‐antimicrobial targeted therapy did not significantly vary between different cohorts according to time from graduation.

**Table 4:** Distribution of participants by year they began working

When started working	Proportion of participants (%)
2020 to 2023	17
2010 to 2019	40
2000 to 2009	15
1990 to 1999	25
1980 to 1989	3

Metronidazole was prescribed to manage giardiasis in both species (30/285 dogs, 6/47 cats). The majority of participants (19/36, 53%) used metronidazole as a second‐line treatment where clinical signs had persisted despite fenbendazole treatment. Other rationales for selecting metronidazole in this context included its use as a first‐line treatment (*n* = 7, 19%) and guidelines or studies supporting its use (*n* = 6, 17%). Of the veterinarians that used metronidazole as a first‐line treatment, 86% (6/7) were from the United Kingdom.

**FIG 1:**
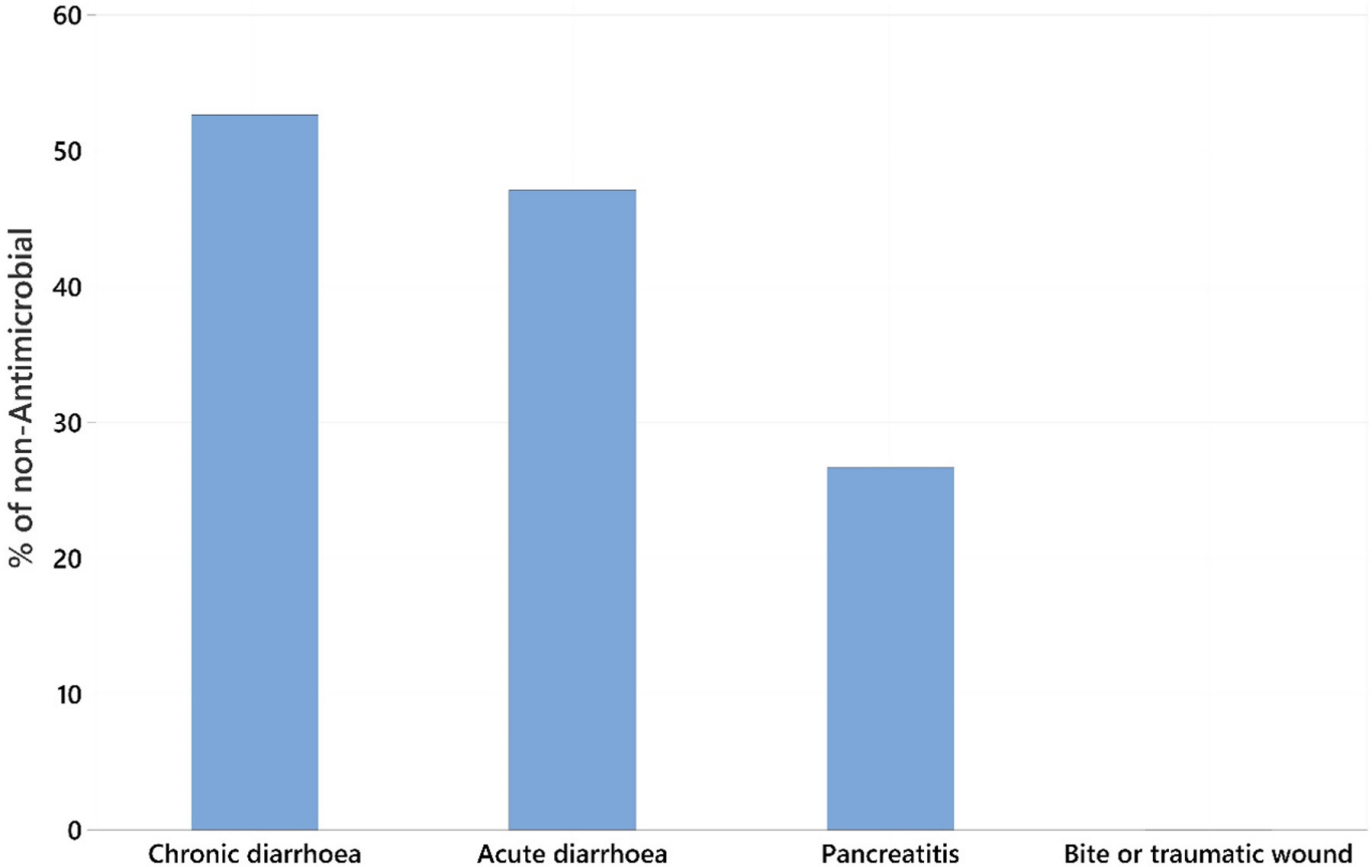
Per cent of condition in which metronidazole was being used for non‐antimicrobial targeted therapy.

The proportion of presentations where metronidazole was used for non‐antimicrobial reasons varied by disease type (Fig 1).

Veterinarians working in referral practice were significantly less likely to use metronidazole for non‐antimicrobial targeted therapy compared to those in first‐opinion practice (3/17, 18% vs. 39/90, 43%, respectively; P = 0.047). Cultures were submitted and the drug was used before culture results were available in 4 cases where a non‐antimicrobial targeted therapy rationale was later cited. There was no significant difference in the likelihood of identifying bacteria by either cytology (P = 0.095) or faecal testing (P = 0.141) when metronidazole was used for non‐antimicrobial targeted therapy compared to its use as an antimicrobial.

In cases of acute diarrhoea, there was no significant difference (P = 0.728) in the rate of probiotics prescribed before metronidazole, whether metronidazole was used as an antimicrobial (30/72, 42%) or for non‐antimicrobial targeted therapy (24/62, 39%). The number of days of diarrhoea prior to starting treatment with metronidazole was significantly higher (P = 0.006) when metronidazole was used as an antimicrobial (median 4 days; IQR 3 to 6) compared to when it was not (median 3 days; IQR 1 to 5).

Metronidazole was used at a median dose and frequency of 12.5 mg/kg (IQR 8.7 to 17.5) and q12h (IQR q12 to q12), respectively, with no significant difference (P = 0.892) between the dose used for antimicrobial and non‐antimicrobial targeted therapy. However, the duration of treatment prescribed was significantly longer when metronidazole was used as an antimicrobial (median 7 days; IQR 5 to 10) compared to its use for non‐antimicrobial targeted therapy (median 5 days; IQR 5 to 7; P = 0.008).

### Outcome

Animals were reported by participants as improved when metronidazole was used in 88% (219/250) cases with no significant difference (P = 0.227) when used as an antimicrobial (131/146, 90%) or for non‐antimicrobial targeted therapy (88/104, 85%*)*. There was no significant difference (P = 0.469) in cases of acute (108/156, 69%) versus chronic diarrhoea (51/79, 65%) that were reported to have improved. Where an opinion was given, positive treatment outcomes were attributed to the use of metronidazole when used as an antimicrobial in 94% (84/89) of cases, compared with 78% (39/50) when it was not (P = 0.004).

Where a specific anti‐inflammatory/immunomodulatory action was described, educational resources (41/92, 45%), team‐based collaboration (29/92, 32%) and specialist consultation (10/92, 11%) were the most commonly cited sources of this rationale. Among this group, 76% (69/91) either agreed or strongly agreed that they knew of other veterinarians who used antibiotics for anti‐inflammatory/immunomodulatory purposes. Other antibiotics (*n* = 29) reported to be used by practitioners for anti‐inflammatory/immunomodulatory action included doxycycline (*n* = 12, 41%), azithromycin (*n* = 7, 24%), oxytetracycline (*n* = 6, 21%), tylosin (*n* = 3, 10%) and sulfasalazine (*n* = 1, 3%).

## DISCUSSION

The present study found that 42% of metronidazole prescribed was selected for non‐antimicrobial targeted therapy, primarily for putative anti‐inflammatory and immunomodulatory effects. A previous retrospective study (Beaudoin et al., 2023) similarly highlighted the use of antibiotics in companion animals for perceived non‐antibacterial benefits. In that study (Beaudoin et al., 2023), antimicrobial intent was described in only 37% of cases, suggesting that veterinarians view metronidazole as a multi‐purpose medication.

### Use of metronidazole in specific conditions

In cases of giardiasis, metronidazole was prescribed in both species, typically as a second‐line therapy after perceived treatment failure of fenbendazole. This protozoal infection has a predominantly commensal role in cats (Barrera et al., 2024; Gruffydd‐Jones et al., 2013) questioning the need for antimicrobial treatment. Even if a role as an enteropathogen is expected, current therapeutic recommendations consistently favour fenbendazole use (ESCCAP, 2025) as resistance to this anthelmintic is considered very rare (Ciuca et al., 2021). The use of metronidazole in this context is discouraged in antimicrobial guidelines (BSAVA/SAMSoc, 2024) as it is more likely to contribute to worsening gastrointestinal dysbiosis, negatively affecting the gut microbiome and metabolome, which may favour persistent giardiasis (Pilla et al., 2020). Additionally, it was unclear whether the giardiasis cases indeed had giardiasis (as opposed to an incidental finding in dogs with diarrhoea of other aetiologies). Notably metronidazole is licensed for the treatment of giardiasis in the United States (FDA 2023).

The UK veterinary antibiotic resistance and sale surveillance data (UK‐VARSS, 2023) demonstrated that metronidazole accounts for 4 to 7% of all antibiotic use (daily defined doses per animal) in dogs and cats and is frequently the most commonly used antimicrobial for gastrointestinal disease (Ellis et al., 2023; Fenimore et al., 2017; Holden & Brennan, 2021; Langlois et al., 2020; Shmalberg et al., 2019). The current study found that gastrointestinal disease was one of the most commonly reported indications cited for metronidazole use. This is despite repeated and recent guidance not to use the majority of antimicrobials in most cases of diarrhoea. Recent international antimicrobial use guidelines for canine acute diarrhoea (Jessen et al., 2024) strongly recommended that antimicrobial treatment should be reserved for cases of acute diarrhoea that present with severe disease and signs of sepsis. These recommendations were supported by a systematic review of the literature (Scahill et al., 2024) that found no benefit from any antimicrobial therapy in the management of acute diarrhoea. Similarly, Cerquetella et al. (Cerquetella et al., 2020) repositioned the use of antibiotics to manage chronic diarrhoea beyond dietary and corticosteroid trials and ideally based on consistent histopathological evidence of bacterial involvement via gastrointestinal biopsies.

In cases of acute diarrhoea, respondents reported that probiotics were frequently used prior to administration of metronidazole. Practitioners may hope to restore/support the gut microbiota with probiotics (Jugan et al., 2017) or at least seek a benign alternative to avoid antimicrobial use. Interestingly, as the duration of diarrhoea increased, antimicrobial activity was more likely to be cited as the reason for selecting metronidazole, suggesting an expectation of antibiotic responsiveness (Dandrieux, 2016). In contrast, a previous retrospective study showed that antibiotics were less likely to be used the longer diarrhoea had been going on for (German et al., 2010). Shmalberg et al. (Shmalberg et al., 2019) demonstrated that there was no statistical difference in the time to resolution between the probiotics, metronidazole and placebo treatment groups. Additionally, Rudinsky et al. (Rudinsky et al., 2022) showed that the time to resolution for the metronidazole group was statistically longer than for the diet or diet plus psyllium groups. As such, it is not clear if the later introduction of metronidazole would offer a shortening effect to the duration of diarrhoea or whether it would only compound the dysbiosis.

### Diagnostic and dosing considerations

Diagnostic investigations, especially in acute disease, were often limited or absent prior to metronidazole use. Empiric therapy may be more common in gastrointestinal disorders, as the diagnostic yield from faecal testing or abdominal imaging is limited in acute or chronic diarrhoea (Leib et al., 2012) and owners may be reluctant to engage in significant costs, especially in first opinion practice (Kipperman et al., 2017).

Demonstration of faecal enteropathogens in acute or chronic diarrhoea is of questionable value (Marks et al., 2011; Werner et al., 2021) owing to the similar rates of bacterial identification in animals with and without diarrhoea.

Bacterial cultures were submitted in some cases where non‐antimicrobial targeted therapy was described as the reason for metronidazole use. This apparent contradiction may indicate that metronidazole is initially prescribed during a period of diagnostic uncertainty (culture pending) to address a potential bacterial aetiology. Metronidazole may then be preferred over alternative options because the additional properties offer a secondary justification. This apparent duality could encourage clinicians to use metronidazole to cover multiple therapeutic options.

Antibiotics such as macrolides have previously been used at lower doses in people for anti‐inflammatory activity (Huckle et al., 2018), an approach known as “subtherapeutic dosing” to minimise the potential for antibiotic resistance. In contrast, the dosing of metronidazole was consistent across both the antimicrobial and non‐antimicrobial targeted therapy groups. This uniformity suggests that veterinarians do not differentiate between antimicrobial and anti‐inflammatory/immunomodulatory when determining the dose.

The longer courses of metronidazole in cases where it was cited for use as an antimicrobial likely reflects a skewing of the dataset by respondents who cited antimicrobial rationales for conditions that require extended treatment (e.g., tetanus) or receive it (chronic diarrhoea). They may be mistakenly attributing a bacterial target in cases of “antibiotic‐responsive diarrhoea (ARD)”. Historic classification highlighted the role of ARD (Dandrieux, 2016; Hall, 2011), but the proportion of animals that respond to antibiotics is very low (Volkmann et al., 2017). Non‐antibiotic treatments, for example, prebiotics, probiotics or faecal matter transplantation (Schmid & Tolbert, 2024; Winston et al., 2024), hold promise to address the dysbiosis and improve clinical signs without recourse to antibiotics. This has been reflected in a new proposed terminology (Dupouy‐Manescau et al., 2024).

### Clinical outcomes

While animals improved when metronidazole was used both as an antimicrobial and for non‐antimicrobial targeted therapy, veterinarians were significantly less likely to attribute clinical resolution to metronidazole when it was used for the latter. This study invited retrospective evaluation of participant’s decision making and assignment of the rationale for selection. The lower attribution of success to metronidazole when used for non‐antimicrobial targeted therapy may suggest a degree of inherent scepticism regarding such an impact. This observation may reflect: (1) a tendency for participants, through the act of completing the survey, to become more self‐critical and question metronidazole’s purported non‐antimicrobial effects or (2) veterinarians employing metronidazole in this capacity despite lingering doubts about its efficacy and recognising the lack of robust evidence of causality.

### Limitations

Oversight of the case identification process was limited and relied on multiple individuals, potentially leading to case selection bias. Additionally, the survey likely appealed to veterinarians with an active interest in antimicrobial stewardship, who may not represent the wider veterinary population, introducing respondent selection bias. Furthermore, due to the study’s retrospective nature, some veterinarians may have inaccurately reported their reasons for prescribing metronidazole, resulting in recall bias.

For both selection biases, we sought to encourage participation from a broad range of practitioners by disseminating the survey link through a wide variety of channels but were not able to incentivise engagement with the study. As a result, the survey findings may not be reflective of the wider practitioner pool. There was an intention to compare responses based on geographical location but there was insufficient uptake in countries other than the United Kingdom despite attempts to distribute the survey in countries the authors either originated or worked in, including Canada, Singapore, the United States and Germany (even with a translation of the survey into German). This likely reflected the need for an investigator to promote and encourage participation locally.

Some veterinarians may not have remembered the details of their original decision‐making. Such rationales are rarely (if ever) documented in clinical records. However, the survey asked participants to describe why they believed they prescribed metronidazole in that situation. Since we know this was a real clinical scenario encountered by the veterinarian, we hoped their later reflection would closely match their original reasoning.

As part of the assessment for survey validity, both phase I and phase II surveys were designed under the guidance of veterinary colleagues working in primary care (face validity) and experts in the veterinary use of antibiotics (content validity). However, no assessment of survey reliability (test–retest, inter‐rater and internal consistency) was conducted and assessments of other types of validity (construct, criterion, predictive) were not performed. Ahmed and Ishtiaq (Ahmed & Ishtiaq, 2021) illustrate that meticulous attention to various types of validity and reliability enhances a survey’s effectiveness and applicability and thereby are important means to objectively evaluate any survey.

The conditions reported were veterinarian‐based diagnoses, not founded on any standardised definitions. However, this was not a significant issue since treatment decisions were made based on the veterinarian’s clinical judgement.

## CONCLUSION

This study suggests that veterinarians are using metronidazole largely for its non‐antimicrobial properties and frequently in contradiction to antimicrobial use guidelines. Future stewardship programs should readjust guidelines to specifically address this prescribing behaviour. This study also found that 19% of veterinarians selected metronidazole based on a prior positive outcome, either in a similar case or the same patient. This reliance on anecdotal reasoning aligns with previous qualitative research involving practitioner interviews (Tompson et al., 2021), which underscored the frequent reliance on such reasoning in driving antibiotic use in companion animals. Such practices may inadvertently lead to inappropriate antimicrobial use, contributing to the development of resistance.

Additionally, factors such as owner expectations were identified as influencing antibiotic selection in 9% of cases in the present study, consistent with findings from Tompson et al. (Tompson et al., 2021). To address these challenges, alternative strategies should be prioritised, including advocating for non‐prescription forms of treatment (BSAVA/SAMSoc, 2018; Spurling et al., 2017) and utilising educational tools such as antimicrobial resistance engagement animations (Wright et al., 2024).

Anti‐inflammatory/immunomodulatory properties have been articulated for certain antibiotics in people. Various inflammatory and immune‐mediated conditions may be managed with antibiotics, including cotrimoxazole in rheumatoid arthritis (Rozin et al., 2001), macrolide antibiotics in chronic inflammatory airway disease (Cameron et al., 2012; Huckle et al., 2018) and tetracyclines in ophthalmic disease (Federici, 2011), despite the absence of a primary bacterial target. In contrast, evidence supporting an anti‐inflammatory/immunomodulatory mechanism of action in dogs and cats is limited.

Dissemination of revised guidelines with an emphasis on veterinarians citing anti‐inflammatory/immunomodulatory properties as their rationale for prescribing metronidazole, may be particularly beneficial for several reasons: (1) educational resources are their most commonly referenced source for anti‐inflammatory/immunomodulatory information, suggesting they may be receptive to updated materials; (2) many of these veterinarians are aware of colleagues who also prescribe antibiotics for anti‐inflammatory/immunomodulatory purposes, providing a potential network for the distribution of new guidelines and (3) since this group commonly uses alternative antibiotics for similar purposes, reducing metronidazole misuse may also help curtail the inappropriate use of other antibiotics for non‐antimicrobial indications.

### Author contributions

**J. Ng:** conceptualisation (equal); data curation (equal); formal analysis (equal); funding acquisition (equal); investigation (lead); methodology (equal); visualisation (lead); writing – original draft (equal); writing – review and editing (equal). **N. Steffensen:** conceptualisation (equal); writing – methodology (equal); validation (equal); review & editing (equal). **I. Battersby:** conceptualisation (equal); validation (equal); writing – review& editing (equal). **J. S. Weese:** conceptualisation (equal); validation(equal); writing – review & editing (equal). **D. Timofte:** writing – review& editing (equal). **P. L. Toutain:** conceptualisation (equal); investigation(supporting); validation (equal); writing – review & editing (equal). **J. L. Granick:** conceptualisation (equal); validation (equal); writing – review& editing (equal). **J. Elliott:** validation (equal); writing – review& editing (equal). **S. Choi:** investigation (supporting); writing – review& editing (equal). **T. Sparks:** data curation (equal); formal analysis(equal); visualisation (supporting); writing – review & editing (equal). **S. Tavener:** conceptualisation (equal); validation (equal); writing – review& editing (equal). **F. Allerton:** conceptualisation (equal); formalanalysis (equal); funding acquisition (equal); investigation (supporting); methodology(equal); supervision (lead); validation (equal); writing – original draft (equal); writing – review and editing (equal).

### Funding information

The organisations did not have any role in the study’s design, implementation or analysis.

### Conflict of interest

No conflicts of interest have been declared.

## Supporting information


Tables S1–S2.

## Data Availability

The data that support the findings of this study are available from the corresponding author upon reasonable request.
