# Supplementary material for: Understanding the rationale for metronidazole use in dogs and cats
Source: J Small Anim Pract. 2025 Jun 30;66(12):909–17. doi: 10.1111/jsap.13910 (PMC12686258; doi:10.1111/jsap.13910)
Supplement: Supplementary file 1 — Tables S1–S2. [file JSAP-66-909-s001.docx]

**Supplementary materials**

| **Breed** | **Number** |
| --- | --- |
| Cross breed | 46 |
| Labrador Retriever | 32 |
| Cocker Spaniel (English) | 15 |
| Golden Retriever | 14 |
| Jack Russell Terrier | 12 |
| Border Collie | 10 |
| Yorkshire Terrier | 9 |
| English Springer Spaniel | 8 |
| Border Terrier | 7 |
| German Shepherd | 7 |
| Miniature Schnauzer | 7 |
| Shih Tzu | 7 |
| West Highland White Terrier | 7 |
| Dachsund | 5 |
| Boxer | 4 |
| Bulldog | 4 |
| French Bulldog | 4 |
| Rottweiler | 4 |
| Whippet | 4 |
| Bedlington Terrier | 3 |
| Chihuahua | 3 |
| Maltese Terrier | 3 |
| Poodle (Standard) | 3 |
| Siberian Husky | 3 |
| Akita | 2 |
| American Staffordshire Terrier | 2 |
| Beagle | 2 |
| Belgian Shepherd (Malinois) | 2 |
| Bichon Frise | 2 |
| Cavalier King Charles Spaniel | 2 |
| Doberman | 2 |
| Greyhound | 2 |
| Pomeranian | 2 |
| Poodle (Miniature) | 2 |
| Pug | 2 |
| Alaskan Malamute | 1 |
| Australian Shepherd | 1 |
| Basset Hound | 1 |
| Borzoi | 1 |
| Cairn Terrier | 1 |
| Chinese Crested | 1 |
| Chow Chow | 1 |
| Cocker Spaniel (American) | 1 |
| Collie | 1 |
| Flat-coated Retriever | 1 |
| Fox Terrier | 1 |
| Havanese | 1 |
| Irish Setter | 1 |
| Irish Terrier | 1 |
| Japanese Spitz | 1 |
| Kerry Blue Terrier | 1 |
| Lakeland Terrier | 1 |
| Lhasa Apso | 1 |
| Parson Russell Terrier | 1 |
| Patterdale Terrier | 1 |
| Pit Bull | 1 |
| Portuguese Water dog | 1 |
| Rhodesian Ridgeback | 1 |
| Staffordshire Bull Terrier | 1 |
| Tibetan Terrier | 1 |
| Trail hound | 1 |
| Welsh Corgi | 1 |
| Unknown | 15 |

**Table 1**: Dog breeds

| **Breed** | **Number** |
| --- | --- |
| Domestic Shorthair | 25 |
| Maine Coon | 5 |
| Ragdoll | 5 |
| Domestic Longhair | 3 |
| British Shorthair | 2 |
| Siamese | 2 |
| Birman | 1 |
| Norwegian Forest | 1 |
| Persian | 1 |
| Unknown | 2 |

**Table 2**: Cat Breeds
